# Optimal Use of Biomarkers in Oncology

**DOI:** 10.1155/2015/423159

**Published:** 2015-08-04

**Authors:** Marie Karlikova, Ondrej Topolcan, Olive T. J. Wolfe, Vivian Barak, Tomas Zima

**Affiliations:** ^1^Central Laboratory of Immunoanalysis, Faculty Hospital in Pilsen and Faculty of Medicine in Plzen, Charles University in Prague, 300 00 Pilsen, Czech Republic; ^2^Biomedical Center, Faculty of Medicine in Pilsen, Charles University in Prague, 323 00 Pilsen, Czech Republic; ^3^Clinical Consultants, Inc., Glen Rock, NJ 07452, USA; ^4^Hadassah Medical Center, Hebrew University, 911 20 Jerusalem, Israel; ^5^First Faculty of Medicine, Charles University in Prague, 121 08 Prague, Czech Republic

The use of biomarkers in oncology has been much more extensive than in any other diseases for more than 30 years. Their use has evolved beyond the research laboratory to more routine clinical use as in diagnosis, monitoring of disease status, and treatment efficacy.

With the advances in molecular biology, other biomarkers than the “classical” serum markers are being developed for clinical use: gene mutations, microRNA, circulating tumor cells, circulating DNA, and others currently in research. Biomarkers such as these are currently being used for prediction: potential “at risk” for disease as well as response to therapy (predictive biomarkers), and for disease prognosis (prognostic biomarkers).

In oncological routine, the correlation of biomarker levels with the clinical status and medical imaging methods is of great significance. The purpose of the correlation is twofold: providing sequential and/or more specific diagnostics and keeping these procedures within affordable economical limits.

This special issue includes a number of reviews and research papers dealing with the above mentioned topics.

*Reviews on Novel Biomarkers*. The advances in molecular research and various “–omics” techniques have enabled us to study a number of potential novel cancer biomarkers. Studying gene expressions and molecular pathways may lead to the discovery of new prognostic biomarkers, markers of early diagnostics, or molecular targets for therapy.

E. Lastraioli et al. present a review on the expression of hERG1 potassium channels in different types of solid cancer (including breast, esophageal, gastric, colorectal, pancreatic, and other cancers). The authors, on the basis of publication review as well as their own findings, argue that hERG1 could be a novel biomarker and point out the overexpression of hERG1 in solid cancers, a feasible determination by immunohistochemistry and the potential of using them as therapy targets as the monoclonal antibody to block them is already available.

M. Rihacek et al. review B-cell activating factor (BAFF), a transmembrane protein, as a biomarker of malignant disease activity and prognostic factor in B-cell derived malignancies such as multiple myeloma. The authors report its proinflammatory properties and its contribution to cancer cachexia. Moreover, BAFF/BAFF-R signaling may be a promising target of future therapy in B-cell derived leukemias and lymphomas.

Cytoglobin, a protein of the globin family, is reviewed by T. Bholah et al. Thanks to the advances in molecular research, a possible role of this protein in cancer has been suggested. In their paper, the authors overview different functions of cytoglobin and provide a perspective on potential research areas that may elucidate its role as cancer biomarker.

The gene for gamma-glutamylcyclotransferase, an enzyme involved in glutathione metabolism, has recently been investigated and reported as an upregulated protein in various cancers. S. Kageyama et al. report an overview of the activities of GGCT in cancer cells.

*Imaging Methods and Oncology*. Imaging methods provide a useful tool in cancer diagnostics and management. Their applications develop in reflection to new technologies and advances in molecular research.

M. Tolia et al. report the value of preirradiated in vivo magnetic resonance spectroscopy parameters in predicting recurrence free survival for patients with high grade gliomas, who undergone postoperative radiotherapy. R. Fusco et al. present the evaluation of the diagnostic value of an imaging protocol that combines dynamic contrast-enhanced MRI (DCE-MRI) and diffusion-weighted imaging (DWI) in patients with suspicious breast lesions. In addition, further research sought to determine if additional information provided by DWI could improve the diagnostic value of breast MRI.

*Predictive Biomarkers and Risk Factors*. J. H. Kim and S. K. Hong report a review, based on meta-analysis of PubMed publications, on potential novel biomarkers in active surveillance of low-risk prostate cancer. These include %[-2] proPSA and Prostate Health Index (PHI), PCA3, TMPRSS2:ERG, the genomic prostate score (GPS), a panel of four kallikrein markers, and the expression levels of different cell cycle progression (CCP) genes.

Y. Cao et al. review a meta-analysis of 25 independent epidemiological studies on the association between hormonal and reproductive factors and thyroid cancer risk. As the title of their paper suggests, reproductive factors but not hormonal factors are associated with thyroid cancer risk.

R. V. Liubota et al. report risk factors of the invasive breast cancer locoregional recurrence. The authors discovered that the scope of the surgical intervention (breast-conserving surgery (BCS) or radical mastectomy (RME)) does not essentially affect the recurrence appearance frequency or the recurrence-free period duration. However, in the BCS group, risk factors such as the presence of metastases in the regional lymph nodes or the hyperexpression Her/2neu presence increased the frequency of the locoregional breast cancer recurrence appearance.

*Prognostic Biomarkers*. M. Tolia et al. describe a study which aimed to identify whether or not the expression of serum baseline C-reactive protein (CRP) and albumin are related to overall survival in non-small-cell lung cancer (NSCLC). The study results suggested that, in fact, high pretreatment CRP and low albumin serum levels were promising independent prognostic factors of overall survival in NSCLC.

*Biomarkers in Treatment Monitoring*. Several indicators, including tumor markers, are used to monitor whether or not a particular cancer treatment is efficient with tolerable toxicity for patients. However, if a tumor marker has low sensitivity and/or specificity, adding another biomarker to the regimen could potentially enhance the treatment evaluation.

S. Kristiansen et al. present a study that focused on the association between hypermethylated DNA and the tumor markers CA 15-3, CEA, and TPA in serum during monitoring of patients with advanced breast cancer.

*Disease Monitoring and Management*. A combination of biomarkers or a set of tests provide a higher sensitivity and/or specificity than a unique biomarker or test. A. Pouliakis et al. report the evaluation of classification and regression trees for the triage of women at risk for cervical intraepithelial neoplasia. The computer-assisted algorithm was based on cytological HPV DNA typing, HPV mRNA detection, p16 immunocytochemical expression, and age and parous status. The authors proposed a methodology which could dramatically reduce the number of women that would require a colposcopy.

This special issue aims to prove the importance of biomarkers for the individualisation of the approach to an oncological patient and the improvement of his quality of life. Biomarkers in oncology are an important tool for diagnostics, prognosis, and therapy monitoring on condition that there is a clear goal for the biomarkers determination; biomarkers are monitored systematically and are interpreted in collaboration of the laboratory and the clinician. Single shot biomarker determination is useless and can lead to incorrect assumptions.

*Marie Karlikova* *Ondrej Topolcan* *Olive T. J. Wolfe* *Vivian Barak* *Tomas Zima*

